# Impact of expert center endoscopic assessment of confirmed low grade dysplasia in Barrett’s esophagus diagnosed in community hospitals

**DOI:** 10.1055/a-1754-7309

**Published:** 2022-03-31

**Authors:** Esther A. Nieuwenhuis, Sanne N. van Munster, Wouter L. Curvers, Bas L. A. M. Weusten, Lorenza Alvarez Herrero, Auke Bogte, Alaa Alkhalaf, B. Ed Schenk, Arjun D. Koch, Manon C. W. Spaander, Thjon J. Tang, Wouter B. Nagengast, Jessie Westerhof, Martin H. M. G. Houben, Jacques J.G.H.M. Bergman, Erik J. Schoon, Roos E. Pouw, on behalf of the Dutch Barrett Expert Centers

**Affiliations:** 1 Department of Gastroenterology and Hepatology, Amsterdam University Medical Centers, location VUMC, Amsterdam, The Netherlands; 2 Department of Gastroenterology and Hepatology, Catharina Hospital, Eindhoven, The Netherlands; 3 Department of Gastroenterology and Hepatology, Saint Antonius Hospital, Nieuwegein, The Netherlands; 4 Department of Gastroenterology and Hepatology, University Medical Center Utrecht, Utrecht University, Utrecht, The Netherlands; 5 Department of Gastroenterology and Hepatology, Isala Clinics, Zwolle, The Netherlands; 6 Department of Gastroenterology and Hepatology, Erasmus Medical Center, Rotterdam, The Netherlands; 7 Department of Gastroenterology and Hepatology, IJsselland Hospital, Cappelle aan den Ijssel, The Netherlands; 8 Department of Gastroenterology and Hepatology, University Medical Center Groningen, University of Groningen, Groningen, The Netherlands; 9 Department of Gastroenterology and Hepatology, Haga Teaching Hospital, Den Haag, The Netherlands; 10 GROW School for Oncology and Developmental Biology, Maastricht University, Maastricht, The Netherlands; 11 Amsterdam Gastroenterology Endocrinology and Metabolism, Cancer Center Amsterdam, The Netherlands

## Abstract

**Background** The optimal management for patients with low grade dysplasia (LGD) in Barrett’s esophagus (BE) is unclear. According to the Dutch national guideline, all patients with LGD with histological confirmation of the diagnosis by an expert pathologist (i. e. “confirmed LGD”), are referred for a dedicated re-staging endoscopy at an expert center. We aimed to assess the diagnostic value of re-staging endoscopy by an expert endoscopist for patients with confirmed LGD.

**Methods** This retrospective cohort study included all patients with flat BE diagnosed in a community hospital who had confirmed LGD and were referred to one of the nine Barrett Expert Centers (BECs) in the Netherlands. The primary outcome was the proportion of patients with prevalent high grade dysplasia (HGD) or cancer during re-staging in a BEC.

**Results** Of the 248 patients with confirmed LGD, re-staging in the BEC revealed HGD or cancer in 23 % (57/248). In 79 % (45/57), HGD or cancer in a newly detected visible lesion was diagnosed. Of the remaining patients, re-staging in the BEC showed a second diagnosis of confirmed LGD in 68 % (168/248), while the remaining 9 % (23/248) had nondysplastic BE.

**Conclusion** One quarter of patients with apparent flat BE with confirmed LGD diagnosed in a community hospital had prevalent HGD or cancer after re-staging at an expert center. This endorses the advice to refer patients with confirmed LGD, including in the absence of visible lesions, to an expert center for re-staging endoscopy.

## Graphical abstract

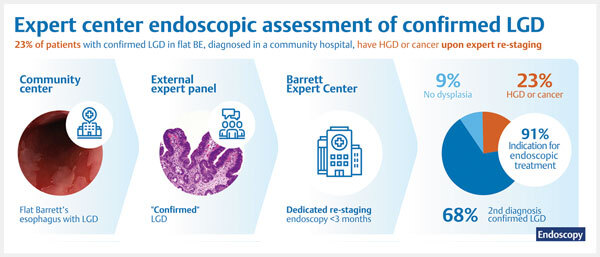

## Introduction

Barrett’s esophagus (BE) is the most important risk factor for development of esophageal adenocarcinoma (EAC). The malignant degeneration occurs through a stepwise process of phenotypic cellular changes: from nondysplastic BE (NDBE), intestinal metaplasia, to low grade dysplasia (LGD), high grade dysplasia (HGD), and eventually EAC 1 . In advanced stages, EAC is a disease with a poor prognosis. Adequate surveillance strategies of patients with BE are therefore essential to detect neoplasia at an early stage when it is amenable to curative endoscopic treatment 2 3 .

The strongest predictor of progression to HGD/EAC in BE is a diagnosis of LGD confirmed by an expert pathologist (i. e. “confirmed LGD”). The histological diagnosis of LGD is challenging because the distinction between dysplastic changes and reactive atypia of reflux-induced inflammation is difficult. Two prior studies demonstrated that LGD diagnosed by a community pathologist, was downgraded to NDBE in 73 %–85 % after review by a BE expert pathologist. After downstaging to NDBE, the risk of progression to HGD/EAC was < 1 % per patient-year 4 5 . In contrast, for confirmed LGD, the risk of malignant progression increased to 9 %–13 % per patient-year 6 7 . Therefore, current guidelines advise that a community diagnosis of LGD is reviewed, and if necessary revised, by an experienced pathologist 8 9 10 11 .

In the Netherlands, BE treatment is centralized. While BE surveillance endoscopies are performed in community hospitals, endoscopic treatment is restricted to nine Barrett Expert Centers (BECs). Patients with visible lesions, HGD, and/or cancer are directly referred to a BEC for endoscopic treatment. Since 2017, the Dutch guideline has recommended that patients with confirmed LGD are also referred to an expert center for a dedicated re-staging endoscopy 8 . This is based on the idea that LGD is a predictor for progression to HGD or cancer and that patients may benefit from dedicated re-staging endoscopies with the option for early intervention if there are visible lesions. Furthermore, several trials have demonstrated significant risk reduction of progression from LGD to HGD/EAC after radiofrequency ablation (RFA) of the BE when compared with surveillance alone 12 13 14 . Most guidelines therefore state that prophylactic ablation should be considered for BE with repetitive diagnoses of LGD 8 9 .

In the current study we evaluated the diagnostic value of re-staging endoscopy performed in an expert center for patients with confirmed LGD.

## Methods

### The BEC registry

All patients referred to a BEC in the Netherlands are registered in a uniform database, (i. e. the BEC registry), which has been described in detail previously 15 . For the current study, we retrospectively reviewed this database. To ensure completeness of data, an additional search of the Nationwide Network and Registry of Histo- and Cytopathology in the Netherlands (i. e. PALGA foundation) was performed. The PALGA database includes all pathology reports in the Netherlands. We selected all patients with confirmed LGD and referral to a BEC from the PALGA database.

### Surveillance for NDBE

Regular surveillance endoscopies for patients with NDBE are performed in community hospitals. Surveillance endoscopies consist of imaging followed by random biopsies according to the Seattle protocol (i. e. 4-quadrant biopsies at every 2 cm) 10 , and targeted biopsies from visible lesions. These biopsy specimens are read by the community hospital pathologist.

Patients with direct indications for treatment (i. e. HGD or worse, and/or a visible lesion) are referred to a BEC. For patients with a diagnosis of LGD assessed by the local pathologist, expert histological review is recommended, and referral to a BEC is advised for cases in which the diagnosis of LGD is confirmed by the expert pathologist.

### Expert panel histopathology revision

A central expert histopathology panel facilitates review of LGD diagnoses. The panel consists of five core pathologists who have been dedicated in the field of BE for at least 15 years and have a median case load of seven cases per week, of which ≥ 25 % are dysplastic 16 17 . Furthermore, all pathologists had participated in the Dutch Barrett Advisory Committee for many years and participated in multiple training programs for endoscopists and pathologists (www.best-acedemia.eu). Nine other BE expert pathologists working in expert centers joined the panel more recently, following quality assessment of 80 indefinite for dysplasia and LGD digital biopsy cases followed by group discussions with the core pathologists 4 . The performance of histopathology revision has been described extensively in a previous publication 16 .

For LGD diagnosed in the Netherlands, biopsy specimens are digitally transferred for review by the panel. The expert panel diagnosis is sent to the endoscopist or pathologist who requested the review.

Upon confirmation of LGD or upstaging to HGD/EAC, the advice is to refer patients to a BEC for a dedicated re-staging endoscopy. Upon downstaging of LGD to indefinite for dysplasia or no dysplasia, patients remain under endoscopic surveillance at the community hospital.

### Barrett Expert Centers

As per the national guideline, within 3–6 months of the diagnosis of LGD, patients are scheduled for a re-staging endoscopy at a BEC 8 . There are nine BECs in the Netherlands, where care is provided by 1–2 experienced pathologists and endoscopists per center; pathologists and endoscopists have participated in joint and specific training programs. Centers adhere to a joint treatment protocol and participate in quarterly meetings to guarantee homogeneity. This infrastructure has been established since 2008, when RFA was adopted for regular clinical care.

Re-staging consists of careful imaging endoscopy with high definition endoscopes with virtual chromoendoscopy. Patients are generally under sedation and most centers schedule dedicated timeslots for BE endoscopies. The Barrett’s segment is described using the Prague C&M classification 18 . Visible lesions are described using the Paris classification 19 and either biopsied or endoscopically resected directly. In addition, random biopsies following the Seattle protocol are taken from the flat Barrett’s segment 20 .

### Endoscopic management

Visible lesions are removed with endoscopic resection techniques. If the specimen shows dysplasia or early cancer, RFA of the remaining BE is generally advised. For flat BE, a diagnosis of HGD or a repeated diagnosis of confirmed LGD during two separate endoscopies (i. e. twice LGD) are indications for prophylactic RFA 12 .

When the re-staging endoscopy shows flat BE with indefinite for dysplasia or no dysplasia, patients are scheduled for surveillance endoscopies in the BEC after 12 months. If no dysplasia is found at these endoscopies, patients are referred to the community hospital and followed up according to the regular NDBE surveillance protocols.

### Study population

We included cases that fulfilled all of the following criteria: 1) flat BE in the absence of visible lesions with LGD detected in a community hospital; 2) confirmed LGD upon expert pathologist review; 3) referral to a BEC between January 2017 and October 2019.

Since 2017, guidelines have advised expert histopathology review including referral to a BEC in cases of confirmation or upstaging to HGD/EAC. Cases with visible lesions assessed in the community hospital were excluded for this study cohort.

### Study endpoints

We defined several endpoints:

- proportion of patients with HGD/cancer or with visible lesions during re-staging in the BEC
- proportion of patients with high risk EAC during re-staging at the BEC, defined as cancer with deep submucosal invasion (i. e. sm2/3), and/or poor differentiation grade, and/or presence of lymphovascular invasion; in contrast, low-risk EAC was defined as any mucosal or superficial submucosal EAC (i. e. ≤ sm1) in the absence of poor differentiation and absence of lymphovascular invasion
- proportion of patients with an indication for (prophylactic) endoscopic treatment upon re-staging; indications for treatment consisted of confirmed LGD at two separate endoscopies, HGD or EAC 8 .

### Statistics

Statistical analysis was performed using the Statistical Software Package IBM SPSS Statistics version 24.0.0.1 for Windows (IBM Corp. Armonk, New York, USA) and R version 3.4.1 for Windows (R Foundation for Statistical Computing, Vienna, Austria. www.R-project.org). Continuous variables were presented as mean with standard deviation (SD) or median with interquartile range (IQR) for normally distributed or skewed data, respectively. Categorical variables were presented as counts with percentages. Adjusted 95 % confidence intervals (CIs) were obtained using simple bootstrapping with 10000 samples. The chi-squared test was performed to compare binary, unpaired results.

### Ethics

The Institutional Review Board of the Amsterdam University Medical Centers declared that the registry was not subject to the Medical Research Involving Human Subjects Act and waived the need for formal ethical review and patient-informed consent. However, written informed consent was obtained for all patients who underwent endoscopic treatment 15 . Patients who had not undergone endoscopic treatment were approached through an opt-out card with the option of declining participation in the study.

## Results

We identified 258 patients with confirmed LGD. In total, 248/258 patients (96 %) were referred to a BEC for a re-staging endoscopy between January 2017 and October 2019 and were included in the analysis. The remaining 10 patients remained in the care of the community hospital and were not referred for varying reasons, including limited life expectancy and/or patient preference.

Baseline characteristics are shown in Table 1 . The majority of patients were male (78 %) and the median age of patients was 69 years (IQR 64–75). A total of 149 patients (60 %) had a history of Barrett’s surveillance at a community hospital for a median duration of 7 years.

**Table 1:** Baseline characteristics.

	All (n = 248)
**Demographics**	
Age, median (IQR), years	69 (64–75)
Male, n (%)	194 (78)
BMI, mean (SD), kg/m ^2^	27 (4)
Smokers 1 , n (%)	
Current	25 (10)
Former	84 (34)
**History**	
History of surveillance prior to referral, n (%)	149 (60)
Duration of prior surveillance, median (IQR)	7 (3–12)
History of LGD prior to referral, n (%)	31 (13)
**Endoscopic BE characteristics**	
Prague classification for length of BE segment, median (IQR), cm	
Circumferential	3 (0–6)
Maximum	5 (3–8)
Hiatal hernia, n (%)	235 (95)
Esophagitis, n (%)	15 (6)
**Visible lesions (n = 58)**	
Paris classification of visible lesions (primary component) 2 , n (%)	
Type 0-IIa	40 (69)
Type 0-IIb	8 (14)
Type 0-IIc	3 (5)
Type 0-Is	1 (2)

IQR, interquartile range; BMI, body mass index; SD, standard deviation; LGD, low grade dysplasia; BE, Barrett’s esophagus.

1 73 (29 %) missing.

2 6 (10 %) missing.

Re-staging endoscopy in the BEC was performed at a median of 3 months (IQR 0–3) after the community hospital endoscopy from which confirmed LGD was diagnosed.

### HGD or cancer during re-staging

In total, 57 patients (23 %) had HGD or cancer during re-staging in the BEC. This included a diagnosis of HGD (32 patients; 13 % [95 %CI 9–18]), low risk EAC (23 patients; 9 % [95 %CI 6–14]), or high risk EAC (2 patients; 1 % [95 %CI 0.01–2]) ( Table 2 ).

**Table 2:** Histopathology findings during re-staging in the Barrett Expert Center.

Diagnosis during re-staging in BEC	Total cohort (N = 248)	No visible lesion detected in BEC 1	Visible lesion detected in BEC
Dysplasia not reproduced, n (%)	23 (9)	22 (96)	1 (4)
New diagnosis of confirmed LGD, n (%)	168 (68)	156 (93)	12 (7)
HGD, n (%)	32 (13)	12 (37)	20 (63)
EAC, n (%)	25 (10)	–	25 (100)
Low risk	23 (9)		
High risk	2 (1)		

BEC, Barrett Expert Center; LGD, low grade dysplasia; HGD, high grade dysplasia; EAC, esophageal adenocarcinoma.

1 Histology based on random biopsies.

**Fig. 1:**
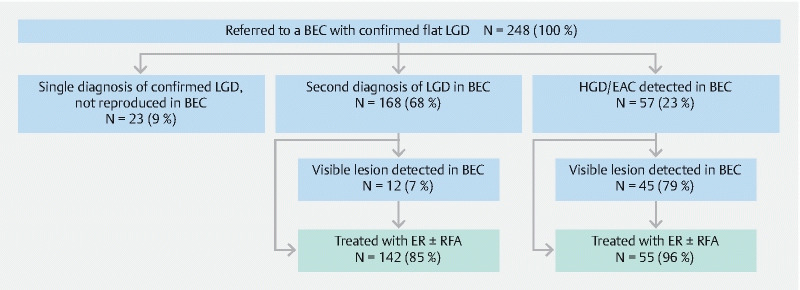
Expert center endoscopic assessment of confirmed low grade dysplasia – patient flow. BEC, Barrett Expert Center; LGD, low grade dysplasia; HGD, high grade dysplasia; EAC, esophageal adenocarcinoma; ER, endoscopic resection; RFA, radiofrequency ablation.

In 168/248 patients (68 %; [95 %CI 62–74]) a second diagnosis of confirmed LGD was found during re-staging at the BEC. In the remaining 23 patients (9 % [95 %CI 6–14]), the initial finding of dysplasia was not reproduced ( Fig. 1 ).

### Visible lesions during re-staging

**Fig. 2:**
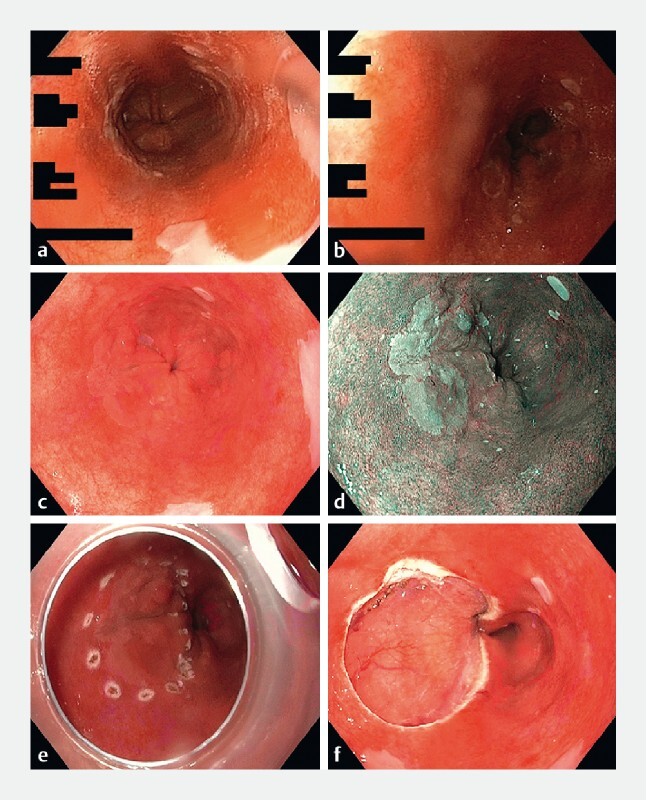
Endoscopic images of a patient referred with confirmed low grade dysplasia (LGD) in random biopsies; no visible lesions were detected at the referring hospital. Images from the community hospital ( **a, b** ) and the Barrett Expert Center (BEC) ( **c–f** ). **a, b** Images in white-light endoscopy (WLE) of a C4M5 Barrett’s segment without signs of reflux esophagitis. The endoscopist reported no visible abnormalities and took random biopsies at three levels (i. e. unclear whether these were taken by following the Seattle protocol). Histopathology analysis showed LGD in all three levels, with p53 expression. Panel review confirmed the diagnosis. **c, d** Images in WLE and narrow-band imaging of the same patient with a Barrett’s segment containing a Paris type 0-IIa visible lesion of 25 mm in diameter, 2 cm above the gastroesophageal junction, at the 7–11 o’clock neutral position. **e** Endoscopic view through the Duette cap: lesion delineated with electrocoagulation markers before starting the endoscopic resection procedure. **f** View of the wound after resection and removal of the cap. Histopathology analysis showed esophageal adenocarcinoma invading the submucosa, with good differentiation, without signs of lymphovascular invasion.

Overall, re-staging in the BEC resulted in detection of a visible lesion in 58/248 patients (23 %). Fig. 2 shows a composite image from a patient with a visible lesion detected at a BEC. Stratified for worst pathology found during re-staging, all 25 patients with EAC were diagnosed with a visible lesion (100 % [95 %CI 86–100]) ( Table 2 ). For patients diagnosed with HGD, a visible lesion was found in 20/32 (63 %; [95 %CI 44–79]). Among patients with a second diagnosis of confirmed LGD, 12/168 patients (7 %; [95 %CI 4–12]) had a visible lesion. Finally, one patient (4 % [95 %CI 0.1–2]) with NDBE was found to have a visible lesion that appeared suspicious for neoplasia during endoscopy and was removed with endoscopic resection, but the final pathology reading showed no dysplasia.

Overall, 51 /58 patients (88 %) had a flat-type lesion (i. e. type 0-II) according to the Paris classification, most commonly type 0-IIa ( Table 1 ).

### High risk cancer during re-staging

Two patients (2/248; 1 %) were diagnosed with a high risk EAC during re-staging. One patient was found to have a visible lesion upon re-staging in the BEC. The patient had no history of surveillance for BE in the community hospital. The time between the first community hospital endoscopy and re-staging endoscopy in the BEC was 3 months. The endoscopic resection specimen showed a deep submucosal cancer (> 500 µm), with lymphovascular invasion and moderate differentiation, with negative deep resection margins. Additional baseline examinations showed lymph node and distant metastasis.

The second patient, also without BE surveillance history, was found to have a visible lesion upon re-staging and endoscopic submucosal dissection was initiated but prematurely aborted due to deep invasion of the proper muscle layer. Additional baseline examinations showed bone metastasis. Time between first community hospital endoscopy and re-staging was 3 weeks.

### Indication for endoscopic treatment

After re-staging in the BEC, 91 % of patients (225/248; [95 %CI 86–94]) had an indication for endoscopic treatment according to current guidelines. Treatment indications consisted of EAC (n = 25), HGD (n = 32), or two diagnoses of confirmed LGD (n = 168).

### Follow-up after re-staging

#### Endoscopic treatment

All patients with HGD (n = 32) and low risk cancer (n = 23) underwent direct endoscopic treatment. Treatment was also initiated in 142/168 patients with a second diagnosis of confirmed LGD. Complete endoscopic eradication was achieved in the majority of patients with a second diagnosis of confirmed LGD, HGD, or cancer (i. e. 94 % vs. 100 % vs. 86 %, respectively). Treatment outcomes have been described in detail in a separate article 15 .

#### Endoscopic surveillance after a second diagnosis of confirmed LGD

Despite a repeat diagnosis of confirmed LGD, 26/168 patients (15 %) underwent endoscopic surveillance instead of prophylactic RFA owing to limited life expectancy and/or patient preference. Median BE length in this group was C5M6 (IQR C1–8; M4–10). Patients were followed for a median of 15 months (IQR 10–23) with a median of 2 follow-up endoscopies (IQR 1–2).

Two patients progressed to HGD (2/26; 8 %; annual risk 6 %). One patient had HGD at the first follow-up after 6 months. The second patient developed HGD at 42 months after baseline staging, with LGD reproduced during each of the three prior follow-up endoscopies. At the moment of progression to HGD, endoscopic treatment was initiated for both patients, with outcomes pending.

#### Endoscopic surveillance after a single confirmed LGD diagnosis

A finding of dysplasia was not reproduced during re-staging in 23 patients. Patients were followed for a median of 19 months (IQR 12–25) with a median of 1 (IQR 1–2) follow-up endoscopy after restaging. Two patients (2/23; 9 %; annual progression risk 6 %) developed HGD, one after 6 months and the other after 30 months after several diagnoses of confirmed LGD.

Overall, when comparing results from all nine BECs, there was no significant difference between the centers.

## Discussion

We assessed the impact of a dedicated re-staging endoscopy by an expert endoscopist upon a diagnosis of flat BE with LGD confirmed by an expert pathologist. To that end, we included 248 patients who were referred to a BEC in the Netherlands with flat BE and a confirmed LGD diagnosis. In 23 % of patients, prevalent HGD or cancer was found during re-staging. This was diagnosed through targeted sampling from a visible lesion in the majority of patients. Overall, 91 % of patients had an indication for endoscopic treatment after the re-staging endoscopy. Our results suggest that patients with confirmed LGD should undergo a re-staging endoscopy by an expert endoscopist.

It is well known that LGD is a challenging diagnosis and guidelines therefore recommend expert pathologist review for each LGD diagnosis 8 9 10 11 . The differentiation between reactive inflammatory changes and early dysplasia is complex. Prior studies have shown that up to 85 % of LGD diagnoses made in a community hospital, are downstaged to NDBE after expert review 6 7 . Most importantly, LGD that was downstaged to NDBE progressed at an annual rate of < 1 %, comparable to “normal” NDBE, whereas LGD that was confirmed had an annual progression risk of 9 %–13 % 6 7 . Of note, in the current study we selectively included patients with LGD that was confirmed by an expert pathologist.

“Expert pathologists” in the current study were defined as pathologists dedicated in the field of BE with a median case load of seven cases a week, of which ≥ 25 % are dysplastic 16 17 . Moreover, pathologists participated in multiple joint training programs with quality assessments followed by group discussions 4 .

Some comparisons with prior studies can be drawn. The aforementioned two studies that assessed progression risks after confirmed LGD did not report a proportion of HGD/EAC and/or visible lesions detected at re-staging 6 7 . However, steep Kaplan–Meier curves during the first 6 months suggest that HGD/EAC was already present at referral to the expert center 6 7 . In the screening cohort of the SURF study, a randomized intervention study comparing RFA with surveillance for patients with LGD, 20/247 patients (8 %) initially diagnosed with confirmed LGD were found to have HGD or cancer during first re-staging in a BEC 12 . In addition, in a recently published retrospective study, the authors aimed to determine the proportion of prevalent HGD or EAC detected by BE referral units in patients referred from the community with a recent expert-confirmed diagnosis of LGD 21 . Similarly to our study, the authors concluded that worse grades of dysplasia (HGD/EAC) are found in a Barrett’s referral unit after referral for confirmed LGD in approximately a quarter (20/75, 27 %) of patients, plausibly representing prevalent HGD/EAC 21 . We may speculate about several explanations for our findings. First, the quality of the endoscopy in the community hospital is likely to play an important role. This is mainly determined by the quality of imaging and the quality of histological sampling. It is well known that detection of visible lesions in BE is challenging. This is especially the case when exposure to visible lesions is low, as in a surveillance setting, partly due to the subtle appearance of early neoplasia, but mainly because general endoscopists are unfamiliar with the endoscopic appearance of neoplasia, as progression to neoplasia is rare (< 1 % annual risk) 22 23 24 . A prior study compared detection rates of visible lesions in community hospitals and after referral in BECs, and showed that expert endoscopists detected a visible lesion in 87 %, compared with 60 % in the community hospitals ( *P* < 0.01) 25 . However, this study selectively included patients with HGD/EAC. The endoscopists at the expert center may therefore have been biased and were looking for a lesion, knowing that the patient had HGD/EAC.

An American study showed that nearly 25 % of endoscopies performed in patients with BE were not adherent to the Seattle protocol 26 . This finding was confirmed in a recent systematic review showing poor adherence to the Seattle protocol, especially in nonexpert centers and in longer BE segments 27 . Adherence may be low due to increased procedure time or incorrect perception of an individual patient’s risk of neoplastic progression.

A second explanation reflects the quality of the endoscopy at the expert center. Endoscopic examination consists of high definition endoscopy with optical chromoendoscopy by an experienced endoscopist under optimal circumstances, with the majority of patients under sedation and with the use of dedicated timeslots for BE endoscopies.

However, if imaging and sampling may be less accurate in a community hospital, why were these patients with a visible lesion containing HGD or cancer then diagnosed with LGD? It seems unlikely that random biopsies with confirmed LGD in the community hospital were accidentally obtained from the visible lesion, and that these biopsies were then read as LGD but not as HGD or cancer. From a pathophysiological perspective, it may be that patients with HGD or cancer have a larger field defect with dysplastic changes. This large field defect with more widespread dysplastic changes may be easier to pick up with random biopsies than a solitary visible lesion. The current study shows that detection of confirmed LGD, even if the BE is deemed completely flat in a community hospital, defines a cohort with a substantial risk for more advanced histology.

Based on our results, we recommend that patients with confirmed LGD in flat BE diagnosed in a community hospital are referred to an expert center for a dedicated re-staging endoscopy. Most importantly, one quarter of these patients may have a visible lesion with HGD or cancer, and 1 % were even found to have a high risk cancer. If these patients had been treated with RFA in a community hospital due to apparent “flat BE,” this would have been inadequate therapy and the risk for progression to advance disease would be substantial.

On the other hand, if these patients with confirmed LGD had not been referred for re-staging at an expert center, surveillance would have been done after 6 months, with a risk of progression in patients with prevalent HGD/EAC. Moreover, a subtle lesion may also have been missed during the second endoscopy, with additional delay and risk for progression. The Dutch and European BE guidelines recommend that patients with confirmed LGD are referred to an expert center for re-staging within 3 months, whereas US guidelines advise re-staging after 3–6 months with high definition and (optical) chromoendoscopy, not necessarily at an expert center 8 9 10 28 29 . Considering the high rates of worse histopathology found at the expert endoscopy, we would advocate for re-staging within 3–6 months upon referral in an expert center as advised by the Dutch and European guideline.

This study has important strengths. This is the first report of a nationwide cohort of BE patients with confirmed LGD who were referred to expert centers for re-staging; the findings have direct implications for clinical care. Our data are homogeneous: all endoscopists and pathologists participated in a specific and joint training program, and all centers followed a uniform treatment and follow-up protocol. We included all patients in the Netherlands who underwent endoscopic re-staging upon confirmed LGD in one of the BECs. We provide high quality data that were collected by dedicated researchers.

We have to address some limitations as well. This was a retrospective study with a risk for selection bias. Most importantly, we could have missed patients with confirmed LGD who were not included in our database. In order to minimize this risk and to ensure complete data, we performed an additional search of the national pathology database, in addition to the BEC registry search. There is also a risk that not all patients with confirmed LGD were referred to an expert center, but only the patients with anticipated high risk for neoplasia, such as those with long BE segments. This would result in an overestimation of the proportion of prevalent HGD in our study. However, as only 10 patients with confirmed LGD were not referred, the effect would be minimal. Finally, although guidelines recommend confirmation of each LGD diagnosis, some endoscopists may have chosen not to apply for pathology review. If specifically those patients with an assumed low risk for prevalent HGD, such as patients with short segment BE, were not referred for pathology review, then again the reported rate for prevalent HGD would overestimate the actual rate. However, our study outcomes do reflect current clinical care and therefore recommendations still hold.

In a minority of community hospital LGD cases (15 %), pathological review was performed by one local expert pathologist instead of review by the panel upon referral, because panel review is advisable, but not mandatory, according to the Dutch guideline 8 . As the endoscopists in the BEC were informed about the presence of LGD in advance, inspection may have been even more meticulous and the threshold to resect visible lesions may have been lower. However, instead of this being a limitation or bias, we feel that this reflects real-life clinical practice and only supports the advice to refer patients with confirmed LGD to an expert center for re-inspection. Unfortunately, we have no data on adherence to the Seattle protocol in the community hospitals. Therefore, we could not draw any conclusions regarding adherence to the Seattle biopsy protocol or possible sampling error. Follow-up data for confirmed LGD that was not treated in our study may be prone to confounding by indication. Downstaging to NDBE during re-staging may either indicate actual downstaging, but more likely reflects sampling error of focal LGD area(s), but it is impossible to differentiate between these two scenarios for patients in the current study. Unfortunately, we had no data on type of endoscope and use of optical chromoendoscopy. Finally, data may be less generalizable worldwide, owing to our homogeneous care setting in the Netherlands.

Our study shows that re-staging by an expert endoscopist upon confirmed LGD is valuable, as a quarter of the patients had prevalent HGD or cancer. Furthermore, 91 % of these patients had an indication for endoscopic treatment upon re-staging. Confirmed LGD entails a high risk of synchronous worse histopathology that can easily be overlooked by inexperienced endoscopists. We advocate for expert endoscopy for all patients with confirmed LGD.
