# Correction: Impact of expert center endoscopic assessment of confirmed low grade dysplasia in Barrett’s esophagus diagnosed in community hospitals

**DOI:** 10.1055/a-1914-2490

**Published:** 2022-08-05

**Authors:** Esther A. Nieuwenhuis, Sanne N. van Munster, Wouter L. Curvers, Bas L. A. M. Weusten, Lorenza Alvarez Herrero, Auke Bogte, Alaa Alkhalaf, B. Ed Schenk, Arjun D. Koch, Manon C. W. Spaander, Thjon J. Tang, Wouter B. Nagengast, Jessie Westerhof, Martin H. M. G. Houben, Jacques J.G.H.M. Bergman, Erik J. Schoon, Roos E. Pouw, on behalf of the Dutch Barrett Expert Centers

**Affiliations:** 1 Department of Gastroenterology and Hepatology, Amsterdam University Medical Centers, location VUMC, Amsterdam, The Netherlands; 2 Department of Gastroenterology and Hepatology, Catharina Hospital, Eindhoven, The Netherlands; 3 Department of Gastroenterology and Hepatology, Saint Antonius Hospital, Nieuwegein, The Netherlands; 4 Department of Gastroenterology and Hepatology, University Medical Center Utrecht, Utrecht University, Utrecht, The Netherlands; 5 Department of Gastroenterology and Hepatology, Isala Clinics, Zwolle, The Netherlands; 6 Department of Gastroenterology and Hepatology, Erasmus Medical Center, Rotterdam, The Netherlands; 7 Department of Gastroenterology and Hepatology, IJsselland Hospital, Cappelle aan den Ijssel, The Netherlands; 8 Department of Gastroenterology and Hepatology, University Medical Center Groningen, University of Groningen, Groningen, The Netherlands; 9 Department of Gastroenterology and Hepatology, Haga Teaching Hospital, Den Haag, The Netherlands; 10 GROW School for Oncology and Developmental Biology, Maastricht University, Maastricht, The Netherlands; 11 Amsterdam Gastroenterology Endocrinology and Metabolism, Cancer Center Amsterdam, The Netherlands

**Correction**

**Impact of expert center endoscopic assessment of confirmed low grade dysplasia in Barrett’s esophagus diagnosed in community hospitals** Nieuwenhuis EA, van Munster SN, Curvers WL et al. Endoscopy, DOI: 110.1055/a-1754-7309 In the above-mentioned article, the title has been corrected. Correct is: Impact of expert center endoscopic assessment of confirmed low grade dysplasia in Barrett’s esophagus diagnosed in community hospitals.. This was corrected in the online version on August 5, 2022.

